# vIRA Inhibition of Antiviral Necroptosis and RIPK3 Binding Are Separable Events

**DOI:** 10.3390/pathogens15010079

**Published:** 2026-01-10

**Authors:** Katherine B. Ragan, Haripriya Sridharan, Aaron S. Stark, Kaela Ilami, Amanda D. Fisher, Olivia N. Brahms, William J. Kaiser, Jason W. Upton

**Affiliations:** 1 Department of Molecular Biosciences, Institute for Cellular and Molecular Biology, LaMontagne Center for Infectious Disease, University of Texas at Austin, Austin, TX 78712, USA; thekatieragan@gmail.com (K.B.R.); haripriya.sridharan@thermofisher.com (H.S.);; 2 Independent Researcher, Atlanta, GA 30317, USA; 3 Thermo Fisher Scientific, Whitefield, Bangalore 560066, India; 4 Department of Biological Sciences, Auburn University, Auburn, AL 36849, USA; aaron.stark.bsc@gmail.com (A.S.S.); onb0003@auburn.edu (O.N.B.); 5 Independent Researcher, New Braunfels, TX 78130, USA; 6 Independent Researcher, Austin, TX 78724, USA; 7 Department of Microbiology, Immunology, and Molecular Genetics, University of Texas Health at San Antonio, San Antonio, TX 78229, USA; fisheradf@gmail.com (A.D.F.);; 8 Independent Researcher, Atlanta, GA 30052, USA; 9 Independent Researcher, Sylvania, GA 30467, USA

**Keywords:** RHIM, RIPK3, MCMV, vIRA, necroptosis

## Abstract

Necroptosis is an antiviral form of programmed cell death modulated by proteins that interact via RIP Homotypic Interaction Motifs (RHIMs). The result of the signaling pathways depends on which RHIM-containing proteins are involved: although both host and viral proteins contain RHIMs, virally encoded RHIM proteins, such as murine cytomegalovirus (MCMV)-encoded viral inhibitor of RIP activation (vIRA) serve to prevent cell death. Although every RHIM contains the same core four-amino-acid pattern, there are variations in individual sequences that we hypothesized would determine the differential outcomes in necroptotic signaling. As such, we replaced the RHIM in vIRA with the RHIMs from other proteins involved in the signaling cascade (RIPK1, RIPK3, ZBP1, ICP6) to assess the effect on necroptosis during MCMV infection. Although these RHIM-swap vIRA constructs remained able to bind to RIPK3, in the context of MCMV infection, they lost the ability to prevent necroptosis. These results are consistent with other studies that demonstrate that RHIM-containing proteins form amyloid fibrils unique to the proteins interfacing. Our results provide biological context for the growing model that the outcome of RHIM-based signaling is influenced by the specific amyloid fibril structures that are driven by the unique amino-acid sequences of each RHIM present.

## 1. Introduction

Programmed cell death provides an avenue by which multicellular organisms clear infections. Cells sense the presence of the pathogen and initiate signaling cascades to thwart the spread of the intruding pathogen to neighboring cells, thus restricting the infection [1]. Necroptosis is an important programmed cell-death pathway that is triggered in response to a variety of viral infections as well as ischemia reperfusion injury, cancer, and neurodegeneration [2]. First recognized as a true cell-death program in 2003, necroptosis is triggered by several types of stimuli sensed by specific pattern-recognition receptors (PRRs) that serve as upstream activators for the lynchpin of necroptotic signaling, receptor interacting protein kinase 3 (RIPK3) [3,4,5,6]. Once it receives the signal from its upstream activator, RIPK3 oligomerizes via its RIP homotypic interaction motif (RHIM) and auto-phosphorylates, prompting its interaction with and phosphorylation of its downstream effector, mixed lineage kinase-like protein (MLKL) [7,8,9,10,11]. Upon phosphorylation, MLKL undergoes a conformational change that causes its N-terminus to bind membrane lipids and disrupt membrane integrity, resulting in loss of osmotic control and cell lysis [10,12]. The signaling events responsible for RIPK3 activation are mediated by upstream adaptor proteins that contain their own RHIMs [1]. Activation of RIPK3 by these adaptor proteins requires intact RHIMs present in both the adaptor and RIPK3 since the interaction between the proteins is mediated by the RHIMs [1].

The classical pathway of necroptosis involves the activation of RIPK3 by its sister kinase RIPK1 in response to activation of death receptors via death ligands, such as TNFα, but necroptosis can also be initiated in response to Toll-like receptor (TLR) signaling as well as viral infection [1]. For example, sensing of pathogen-associated molecular patterns (PAMPs), such as double stranded RNA (dsRNA), by TLR3 causes TIR domain-containing adapter-inducing interferon-β (TRIF) to participate in a RHIM–RHIM interaction with RIPK3, resulting in RIPK3 activation [1,13,14]. In response to a variety of viral infections (herpesviruses (murine cytomegalovirus (MCMV), herpes simplex virus 1 (HSV1), varicella zoster virus (VZV)), influenza A virus (IAV), and vaccinia virus), Z-DNA binding protein 1 (ZBP1/DAI/DLM1) is responsible for sensing infection and interacting with RIPK3 [15,16]. ZBP1 contains two RHIM-like sequences: RHIM A is responsible for interacting with and thus activating RIPK3, while RHIM B has been implicated in interacting with RIPK1 and initiating the NFκB response [17,18].

In the ongoing battle between hosts and pathogens, viruses have evolved proteins containing their own RHIMs that serve to manipulate and attempt to impede the host immune response [16,19,20,21]. The most well-characterized example is the viral inhibitor of RIP activation (vIRA) encoded by the M45 gene of MCMV. vIRA is the large subunit of the ribonucleotide reductase (R1) for MCMV and has an N-terminal RHIM domain and a catalytically inactive ribonucleotide reductase (RR) domain in its C-terminus [19,22]. Following MCMV infection, vIRA binds to RIPK3 via RHIM–RHIM interaction, inhibiting the necroptotic cascade by preventing the downstream activation of MLKL [1,19]. We hypothesize that vIRA functions by sequestering RIPK3 through RHIM-RHIM interactions, thus preventing ZBP1 from activating RIPK3 and/or preventing RIPK3 from phosphorylating MLKL [1,19]. Additionally, HSV-1 encodes a RHIM-containing catalytically active R1 protein known as ICP6 [23]. ICP6 can function either as a pro- or anti-viral protein, in a species-dependent manner. During HSV-1 infection of human cells, ICP6 inhibits ZBP1-dependent RIPK3 necroptosis similarly to vIRA by binding RIPK3 [21]. In contrast, in murine HSV-1 infected cells, ICP6 functions as a driver of necroptosis by nucleating RIPK3 oligomers, resulting in RIPK3 phosphorylating MLKL and initiating necroptotic cell death [24,25]. In the context of the TNFα/RIPK1 axis of necroptosis, vIRA inhibits death in both mouse and human cells, whereas ICP6 only provides protection from cell death in human cells [19,25].

RHIMs are highly conserved sixteen-amino-acid sequences possessing a core four amino acids consisting of a sequence of a nonpolar amino-acid-glutatmate-non-polar amino-acid-glycine (XQXG) [26]. RHIM–RHIM interactions require this XQXG sequence; mutating XQXG to a tetra-alanine (AAAA) repeat completely abrogates binding between RHIM-containing proteins [26]. However, an exact difference in RHIM sequence that correlates with either pro- or anti-viral function remains to be determined. Recent studies have probed the structures of both human and murine RHIM-containing proteins to elucidate what it is that makes a RHIM pro- or anti-viral. Solid-state NMR (ssNMR) and cryogenic electron microscopy (cryo-EM) revealed that RIPK1, RIPK3, and vIRA form amyloid fibrils when they complex with each other [27,28,29,30,31,32]. Importantly, the structure of these fibrils is determined by which RHIM-containing proteins are present, with additional structural differences observed for homo- versus hetero-amyloids, suggesting that the outcome of RHIM signaling is directly related to the types of fibrils formed. In support of this hypothesis, a vIRA chimera containing the RIPK3 RHIM blocks necroptosis in cells expressing a RIPK3 chimera containing the vIRA RHIM, while cells expressing WT RIPK3 succumb to programmed cell death [29]. Similarly, substituting the RHIM of vIRA into ICP6 (ICP6^M45-RHIM^) results in a protein that provides species-independent protection from both viral-induced and TNFα-induced necroptosis [25]. The reciprocal recombinant protein (substituting the RHIM in vIRA for the RHIM in ICP6 (M45^ICP6-RHIM^)) behaves like WT ICP6, causing necroptosis without stimulation in murine cells while providing protection from necroptosis in human cells [25]. Together, these data suggest that the sequences of both the viral and the host RHIMs present determine the structures of the amyloids formed, which ordains the outcome of RHIM signaling.

Therefore, we hypothesized that the question of pro- or anti-viral characteristics of RHIMs goes beyond the ability to bind RIPK3 and is determined by each unique RHIM participating in the interaction. To address this hypothesis, the sixteen-amino-acid RHIMs of RIPK1, RIPK3, ZBP1-A, ZBP1-B, TRIF, and ICP6, and the putative RHIM of PARP12, were substituted into the sixteen-amino-acid RHIM of vIRA in expression constructs (referred to as M45 RHIM-swap proteins: M45^RIPK1-RHIM^, M45^RIPK3-RHIM^, M45^ZBP1-A-RHIM^, M45^ZBP1-B-RHIM^, M45^TRIF-RHIM^, M45^ICP6-RHIM^, and M45^PARP12-RHIM^). As expected, all true RHIMs in the context of the M45 protein (M45^RIPK1-RHIM^, M45^RIPK3-RHIM^, M45^ZBP1-A-RHIM^, M45^TRIF-RHIM^, and M45^ICP6-RHIM^) were able to bind to RIPK3. The M45 RHIM-swap proteins that were able to bind RIPK3 failed to protect cells from TNFα-induced necroptosis in murine cells, which was expected, given that in murine cells, each of these RHIMs serves to propagate necroptosis rather than inhibit it.

To probe the functionality of M45 RHIM-swap proteins in the context of MCMV infection, we generated recombinant mutants of MCMV expressing the M45 RHIM-swap proteins that bound RIPK3. These viruses were viable and replicated similarly to WT MCMV in cell lines resistant to virus-induced necroptosis but were severely attenuated for replication in cell lines sensitive to necroptotic cell death. Moreover, all M45 RHIM-swap viruses induced ZBP1-dependent necroptosis to levels similar to M45*mut*RHIM MCMV (containing a tetra-alanine mutation in the place of the RHIM) and with similar death kinetics. Together, our data suggest that a model of vIRA/M45 protection from necroptosis relying solely upon RHIM-dependent sequestration of RIPK3 may be overly simplistic, and consideration of intrinsic features encoded within the vIRA RHIM is critical to prevent RIPK3-dependent necroptosis.

## 2. Materials and Methods

### 2.1. Cells and Reagents

HEK293T (ATCC CRL-3216), SVEC4-10 (ATCC CRL-2181), NIH 3T3 (ATCC CRL 1658), and 3T3-SA (ATCC CCL92) cell lines were obtained from ATCC and maintained, as previously described [33]. All cell lines were confirmed and routinely monitored to ensure that they remained mycoplasma-free (LookOut Mycoplasma PCR Detection Kit (MP0035); Sigma-Aldrich, St. Louis, MO, USA). ZBP1 knock out SVEC4-10 cells were generated as previously described [34].

### 2.2. Plasmids, Transfections, Immunoblotting, Antibodies, and Immunoprecipitation

vIRA RHIM-swap constructs were Gibson-cloned into both the EGFP-N1 vector and the GST-N1 vector using the primers listed in Table 1. Transfections and immunoprecipitations were performed as previously described [17]. Immunoblotting was performed as previously described [33]. The following antibodies were used: rabbit anti-RIPK3 (IMG-5523; Imgenex, Centennial, CO, USA), mouse anti-m123/IE1 (CHROMA101; Center for Proteomics, University of Rijeka, Rijeka, Croatia), mouse anti-β-Actin (Clone AC-74; Sigma, St. Louis, MO, USA), mouse anti-FLAG M2-Peroxidase (Clone M2; Sigma-Aldrich, St. Louis, MO, USA), mouse anti-GST-HRP (Clone 8-326, Thermofisher, Rockford, IL, USA), mouse anti-GFP (Clone 4B10, Cell Signaling, Danvers, MA, USA), donkey anti-mouse IgG-HRP (Vector Laboratories, Newark, CA, USA), and donkey anti-rabbit IgG-HRP (Vector Laboratories, Newark, CA, USA). Blots were visualized using ECL Prime Western Blotting Detection Reagent (GE Healthcare, Marlborough, MA, USA) and exposure to film. Digital images were generated with a CanoSCAN LIDE 700F slide/film scanner (Canon, Melville, NY, USA) and images processed with Canvas X16 software (ACD Systems International, Ft. Lauderdale, FL, USA). No digital enhancements were applied. All immunoblot data presented are representative of at least three independent experiments. Immunoprecipitation was performed using Pierce-GST magnetic agarose beads (ThermoFisher, Rockford, IL, USA) according to the manufacturer’s protocol.

### 2.3. Immunofluorescence

HEK293T cells were transfected with the EGFP-N1 M45 RHIM-swap constructs, as described above, for 24 h. Cells were washed 3 times in PBS and fixed with 4% paraformaldehyde for 20 min at room temperature with rocking. Samples were washed 3 times with PBS, then treated with 1.25 μg/mL Hoechst 33342 (ThermoFisher Scientific, Eugene, OR, USA) at 1.25 μg/mL concentration in PBS and visualized on the EVOS-FL cell imaging system.

### 2.4. Generation of RHIM-Swap MCMVs

Recombineering was performed as previously described [33,35]. The M45*mut*RHIM MCMV BAC and M45^SacB/Kan^ bacmid, which contains the levensucrase (SacB), and kanamycin (Kan) gene cassettes in the M45 locus have been previously described [33]. RHIM-containing fragments were amplified from plasmid chimeric RHIM proteins (previously described), with 50 nucleotide overhangs corresponding to the MCMV genome flanking the RHIM of M45 with the primers, HS36 and HS37. PCRs were *DpnI*-treated, gel-purified, and electroporated into temperature-induced DH10B cells containing pSIM6 and M45^SacB/Kan^ bacmid. Recombinants were selected for kanamycin sensitivity and sucrose resistance. The genomic integrity of the BACs was confirmed after each step of recombination by RFLP analysis using *HindIII* and *EcoRI* digest. PCR amplification of the N-terminus containing the RHIM was carried out with the ASP01C and ASP02B primers and confirmed by sequence analysis. The mutated locus of M45 RHIM-swaps were confirmed by PCR amplification of the RHIM locus, using the HS36 and HS37 primers. Oligonucleotide sequences for recombineering are listed in Table 2.

### 2.5. Viruses and Infections

The parental WT and M45*mut*RHIM viruses have been previously described [33]. vIRA RHIM-swap viruses were generated in NIH3T3, as previously described [36]. Viruses were propagated, clarified, and concentrated, and then titered by plaque assay on NIH3T3 cells, as previously described [33].

### 2.6. Cell Viability Assays

Cell viability assay was performed as previously described [33] using Promega Cell Titer Glo (Promega, Madison, WI, USA) kit at 18–22 h post infection. Cell death was also determined by monitoring cell integrity with the live cell impermeant nucleic acid stain—Sytox Green (Invitrogen, Eugene, OR, USA). Cells (10^4^ cells/well) were seeded into Corning 96-well tissue culture plates. After infection for 1 h, cells were cultured in the medium containing 50 nM Sytox Green and 1.25 μg/mL Hoechst 33342 (Thermo Fisher Scientific, Eugene, OR, USA) and imaged on a Citation Cell Imaging Multi-Mode Reader (Biotek, Agilient, Santa Clara, CA, USA). Two images of each well were collected. The numbers of Sytox Green-positive and Hoechst 33342-positive cells per image were calculated (% Cell death = Sytox Green+ cells/Hoechst 33342+cells). Data were analyzed by one-way ANOVA and Dunnett’s multiple comparisons test with single pooled variance.

### 2.7. FACS Assay

SVEC4-10 cells were transiently transfected with GFP M45 RHIM-swap constructs for 48 h. Cells were then treated with 25 ng/mL of murine TNF (PeproTech, Eugene, OR, USA) and 50 μM zVAD-fmk (Enzo life sciences, Farmingdale, NY, USA) for 3 h followed by treatment with 500 nM concentration of propidium iodide (PI) (ThermoFisher Scientific, Eugene, OR, USA). Cells were then subjected to FACS sorting on a BD Accuri SORP Flow Cytometer on the basis of GFP/PI positivity. Viability was determined by the percentage of GFP positive, PI negative cells compared with vehicle-treated samples.

## 3. Results

### 3.1. 1-277 M45 RHIM-Swap Constructs Bind RIPK3 but Do Not Protect from TNFα-Induced Necroptosis

**Figure 1:**
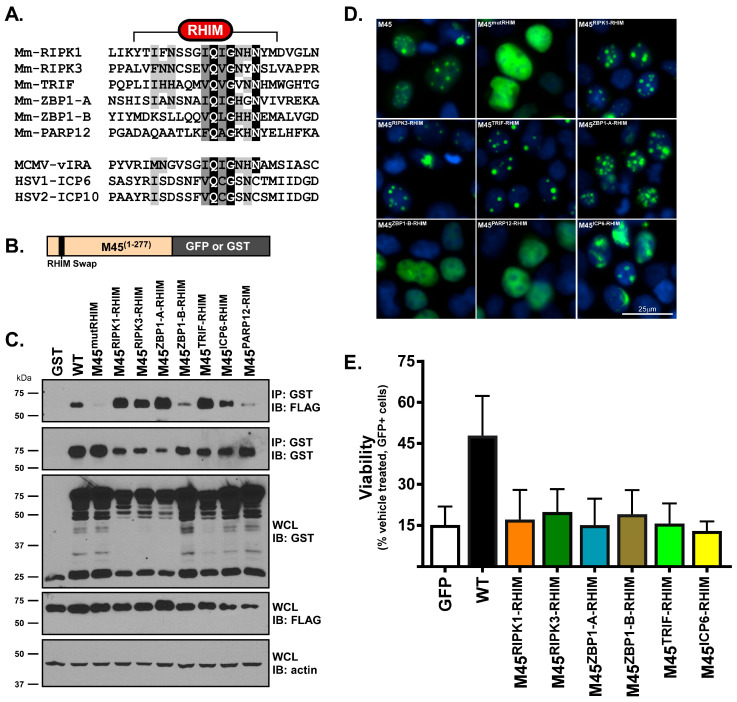
M45(1-277) RHIM-swap constructs bind RIPK3 but do not protect from TNFα-induced necroptosis. (**A**) Alignment of RHIM-containing proteins in this study. Grey bars indicate similar amino acids, black bars indicate identical amino acids. (**B**) Generalized schematic diagram of M45 RHIM-swap fusion proteins. (**C**) HEK293T cells transiently expressing GST-M45 constructs and FLAG-RIP3 were subjected to GST-immunoprecipitation followed by immunoblot analysis for GST and FLAG. β-actin was used as a loading control. (**D**). Immunofluorescence micrographs showing the localization of GFP-M45 RHIM-swap constructs transiently transfected into HEK293T cells. (**E**) Quantification of FACS assay. SVEC4-10 cells transiently expressing GFP-M45 RHIM-swap constructs were treated with TNF for 3 h and stained with propidium iodide (PI). Cells were sorted on the basis of GFP expression and PI permeability and quantified on the basis of GFP positive/PI negative cells divided by total cells. Error bars represent standard deviation (*n* = 3 biological replicates).

Previous work has demonstrated that ICP6^M45-RHIM^ prevents necroptosis in both mouse and human cells like WT M45 [25]. Conversely, the reciprocal substitution, M45^ICP6-RHIM^, protects from necroptosis in human cells while initiating death in murine cells similar to WT ICP6 encoded by HSV1 [25]. This result raised the distinct possibility that the protection from necroptosis offered by WT M45 was due to the RHIM sequence itself (Figure 1A) and that RHIMs may retain their pro- or anti-viral function regardless of protein context. To test this hypothesis, we cloned the core 16 amino acids of RHIM sequences from other RHIM-containing proteins into the core 16 amino-acid position of the N-terminal portion of M45 (amino acids 1-277) with either a C-terminal GST or GFP tag (Figure 1B). The N-terminus of M45 was selected for use in this assay because it is a product of a natural cleavage process during infection [37] that we and others have demonstrated is sufficient to protect from necroptosis [19].

To determine if these M45 RHIM-swap constructs retained their functionality, we assessed their ability to bind to RIPK3 in transiently transfected cells. GST-tagged M45 RHIM-swap constructs were co-transfected with FLAG-tagged RIPK3 into HEK293T cells, and immunoprecipitation followed by immunoblot analysis was performed to assess RIPK3 binding. As expected, each of the RHIMs previously characterized as being “true” RHIMs (RIPK1, RIPK3, ZBP1 RHIM A, TRIF, and ICP6) retained the ability to bind to RIPK3 in the context of the M45 protein, whereas the tetra-alanine mutant RHIM (M45*mut*RHIM) remained unable to bind RIPK3 (Figure 1C, [19]). M45^ZBP1-B-RHIM^, containing the ZBP1 RHIM B that has previously been reported to be unable to bind RIPK3^31^, bound RIPK3 in the context of M45 at minimal levels. M45^PARP12-RHIM^, which contains the putative RHIM sequence in PARP12 [38], bound at similarly minimal levels. Together, these results indicate that vIRA is capable of supporting functional RHIMs from other cellular proteins.

We next examined the localization of M45(1-277) and investigated how these M45 RHIM-swap proteins behaved in cells. HEK293T cells were transiently transfected with the GFP-tagged RHIM-swap constructs for 48 h; this was followed by analysis via fluorescent microscopy (Figure 1D). Transfected WT M45(1-277) localization is nuclear with distinct nuclear puncta. Although M45*mut*RHIM is also nuclear, puncta fail to form, indicating that M45(1-277) is targeted to the nucleus and that the formation of the observed nuclear puncta is RHIM-dependent. Indeed, M45^RIPK1-RHIM^, M45^RIPK3-RHIM^, M45^ZBP1-A-RHIM^, M45^TRIF-RHIM^, and M45^ICP6-RHIM^, all of which retain the ability to bind to RIPK3 (Figure 1C), also formed nuclear puncta similar to those formed by WT M45. Conversely, M45^ZBP1-B-RHIM^ and M45^PARP12-RHIM^ display a dispersed nuclear localization akin to M45*mut*RHIM (Figure 1D). In contrast, M45^ICP6-RHIM^ forms discrete nuclear aggregates but with distinct fiber-like structures in addition to puncta (Figure 1D). While the actual nature of these puncta remains unknown, and their formation may be an unintended consequence of transient overexpression, it is likely that these puncta are indicative of the amyloid fibril structures formed by RHIM–RHIM interactions [27,28,29,30,31,32]. The formation of these puncta also has the potential to serve as a simple screening tool to determine if a putative RHIM does indeed participate in RHIM–RHIM interactions.

To determine which of the M45 RHIM-swaps protects from TNFα-induced necroptosis, we performed a FACs-based cell viability assay using the murine endothelial cell line SVEC4-10, which we and others have previously shown to contain all the machinery required for necroptosis and which are highly sensitive to TNF- and virus-induced necroptosis [33]. Cells were transfected with either an empty vector GFP negative control or a GFP-tagged vIRA RHIM-swap construct for 48 h. Cells were then treated with TNF and zVAD for 3 h to induce necroptosis and stained with propidium iodide (PI); this was followed by cell sorting. Viability was measured as the percentage of the total cells that were GFP positive and PI negative. As expected, compared with the empty vector, WT M45 protects cells from TNFα-induced necroptosis (Figure 1E). Surprisingly, even though M45^RIPK1-RHIM^, M45^RIPK3-RHIM^, M45^-ZBP1-RHIM^, M45^TRIF-RHIM^, and M45^ICP6-RHIM^ can bind RIPK3 (Figure 1C) and form nuclear puncta similar to WT M45 (Figure 1D), these RHIM-swap constructs failed to provide protection to susceptible cells from necroptosis (Figure 1E). Thus, despite being able to bind RIPK3, M45 RHIM-swaps are insufficient to protect cells from TNF-induced necroptosis, suggesting that other specific features of its natural RHIM are necessary for the anti-necroptotic function of M45.

### 3.2. M45 RHIM-Swap Recombinant MCMV Replication Is Restricted in Cell Lines Permissive to Necroptosis

**Figure 2:**
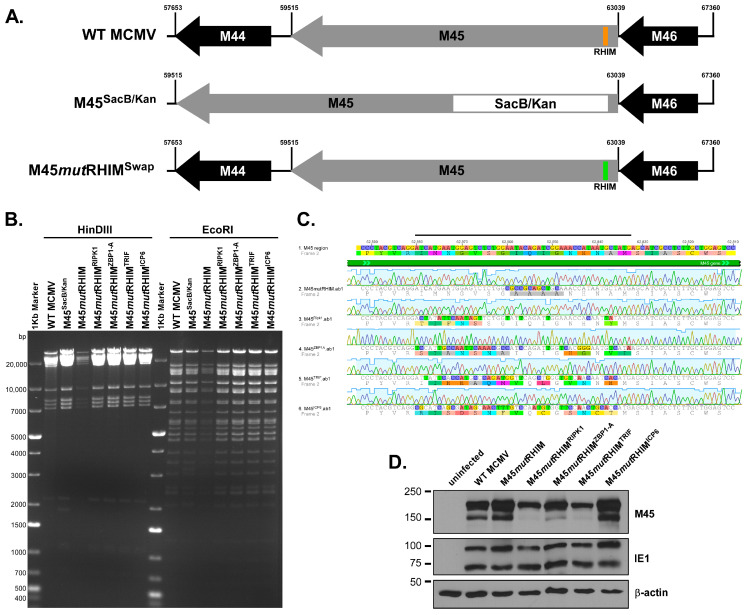
Construction of RHIM-swap viruses. (**A**) Schematic representation of recombineering strategy used to produce RHIM-swap viruses. (**B**) RFLP performed on BACs containing the M45 RHIM-swaps. Enzymes used for digests are indicated, and the ladder marker is NEB 1 kb plus. (**C**) Sequence alignment of PCR amplification over the RHIM from DNA isolated from each RHIM-swap viral preparation. (**D**) Immunoblot of each M45 RHIM-swap virus 24 hpi infected at an MOI of 5 for M45 and IE1. β-actin was used as a loading control.

Given that M45 1-277 RHIM-swap constructs are unable to protect from TNFα-induced necroptosis (Figure 1E) despite being able to bind RIPK3 (Figure 1B), we next wanted to probe the functionality of these RHIM-swap M45 proteins by examining the behavior of the full-length protein in the context of MCMV infection. To do so, recombinant viruses were generated utilizing bacterial artificial chromosomes (BACs), as previously described [33], to replace the 16 amino acids of the M45 RHIM with the RHIMs of RIPK1, ZBP1-A, TRIF, and ICP6 (Figure 2A). These BACs were subjected to restriction fragment length polymorphism analysis to ensure that the MCMV genome remained intact (Figure 2B). Additionally, Sanger sequencing was performed on each virus across the RHIM region to confirm that the intended RHIM-swap mutations were present and that no additional mutations were introduced during the cloning process (Figure 2C).

To demonstrate that these viruses express M45 at equal levels, NIH3T3s were infected with each virus at a multiplicity of infection (MOI) of 5 and lysates were collected 24 h post infection (hpi). Immunoblot analysis was performed to probe for the presence of viral antigens and revealed that all viruses expressed full-length M45 at 24 h post infection as well as equivalent levels of IE1, a marker for MCMV infection (Figure 2D). M45*mut*RHIM^RIPK1^ and M45*mut*RHIM^TRIF^-swap viruses showed a slight deficit in expression of full-length M45, as well as the faster-migrating cleavage product [23], compared to WT (Figure 2D). Whether this is due to protein instability or improper processing remains unclear, each of the recombinant viruses express mutant M45.

**Figure 3:**
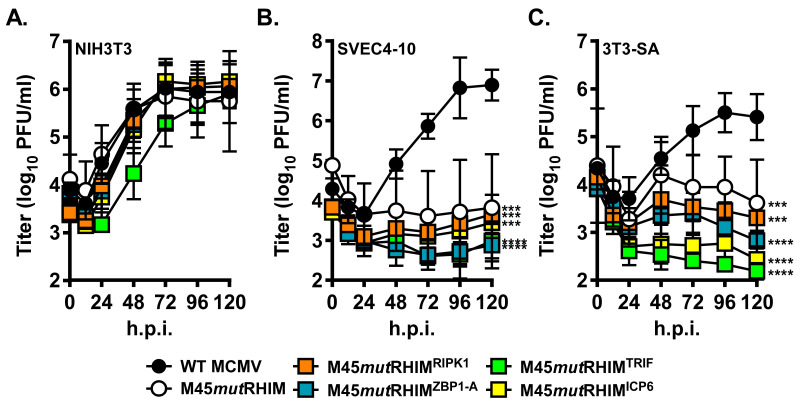
RHIM-swap virus replication is restricted in cell lines permissive to necroptosis. Multi-step growth curve analysis of each M45-RHIM-swap virus (MOI 0.5) in the following cell lines: (**A**) NIH3T3, (**B**) SVEC4-10, and (**C**) 3T3-SA; (*n* = 3–5 biological replicates; *** *p* < 0.001, **** *p* < 0.0001).

To assess the ability of the M45 RHIM-swap viruses to replicate, multi-step growth curve analysis was performed on several cell lines. In NIH3T3s, a cell line permissive to MCMV infection, all viruses replicated to WT MCMV levels as expected (Figure 3A). In contrast, SVEC4-10s show replication of M45 RHIM-swap viruses at remarkably reduced levels: although WT MCMV replicates to high levels during a multi-step growth curve, M45*mut*RHIM MCMV, as well as each M45 RHIM-swap virus, are unable to propagate (Figure 3B). Similarly, 3T3-SAs, a murine fibroblast cell line that expresses high levels of RIPK3 and ZBP1 [39], supported the replication of WT MCMV while restricting the replication of M45*mut*RHIM MCMV and each M45 RHIM-swap MCMV (Figure 3C). Together, these results suggest that M45 RHIM-swap viruses fail to suppress necroptosis during infection of sensitive cells and function similarly to M45*mut*RHIM.

### 3.3. M45 RHIM-Swap MCMVs Do Not Inhibit Virus-Induced ZBP1-Dependent Necroptosis

**Figure 4:**
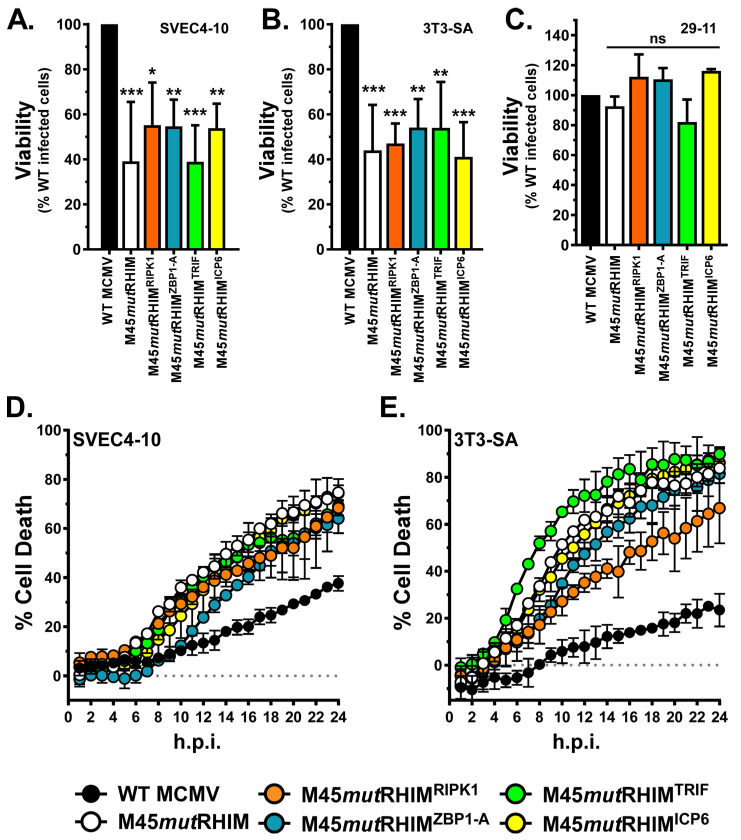
M45 RHIM-swap MCMVs bind RIPK3 but do not inhibit virus-induced necroptosis. Relative end-point (18 h post infection) viability of (**A**) SVEC4-10 cells, (**B**) 3T3-SA cells, and (**C**) 29-11 cells infected with the indicated M45 RHIM-swap MCMV compared to WT MCMV (MOI = 10). Error bars indicate standard deviation from the mean (*n* = 3; * *p* < 0.05, ** *p* < 0.01 *** *p* < 0.001, ns = not significant). (**D**,**E**) Kinetics of cell death of indicated cell lines infected with the indicated M45 RHIM-swap virus (MOI 10), measured in real time by Sytox green incorporation on (**D**) SVEC4-10 cells or (**E**) 3T3-SA cells. Dotted line indicates level of 0% cell death for reference. Error bars indicate standard error (*n* = 3 biological replicates).

We next wanted to compare cell viability between cells infected with WT MCMV, M45*mut*RHIM MCMV, and each M45 RHIM-swap MCMV. To do so, cells were infected with each virus at an MOI of 10 for 18 h, and viability was assessed via an endpoint cell viability assay. Neither SVEC4-10s nor 3T3-SAs undergo necroptosis in response to WT MCMV infection (Figure 4A,B). However, when infected with M45*mut*RHIM MCMV, these cells undergo necroptotic cell death [33] (Figure 4A,B). Infection with each M45 RHIM-swap MCMV results in reduced viability of infected cells (Figure 4A,B). This raised the possibility that M45 RHIM-swap viruses are driving necroptosis, similarly to ICP6-driven necroptosis in murine cells [24,25].

To determine whether this death is due to ZBP1 activation of RIPK3 rather than M45 RHIM-swap proteins nucleating RIPK3, we employed the 29-11 cell line, a SVEC4-10 derivative in which ZBP1 has been knocked out by CRISPR/Cas9 targeting [34]. Unlike WT SVEC4-10s, the 29-11 cells did not undergo necroptosis in response to infection with M45*mut*RHIM MCMV, as expected [34]. When infected with any of the M45 RHIM-swap viruses, the 29-11 cells remained viable, suggesting that the M45 RHIM-swap viruses induce necroptosis via the canonical ZBP1-dependent pathway (Figure 4C).

To further examine the kinetics of these viral infections, real-time kinetic analysis of cell death over a 24 h infection period was performed on both SVEC4-10s and 3T3-SAs. In agreement with our previous data (Figure 4A,B), WT MCMV does not induce significant amounts of cell death, while M45 RHIM-swap viruses induce levels of cell death similar to those of M45*mut*RHIM MCMV (Figure 4D,E). These results are consistent with the replication data (Figure 3) and support the conclusion that M45 RHIM-swap proteins are insufficient to inhibit ZBP1-dependent necroptosis. Together our results indicate that the natural RHIM of vIRA is functionally distinct from other RHIMs.

## 4. Discussion

Here, we show that although 1-277 M45(1-277) RHIM-swap constructs can bind to RIPK3 in a transient transfection, they do not confer protection onto cells sensitive to necroptotic cell death following TNFα treatment, in contrast to WT M45. Since the functionality of virally produced, full-length RHIM-swap proteins had yet to be explored, we built recombinant MCMV strains expressing the M45 RHIM-swap constructs that bind RIPK3 following transfection. Each recombinant virus produced M45 at levels comparable to WT MCMV and was able to replicate to WT MCMV levels in NIH3T3s, a cell line permissive to MCMV infection. However, in cell lines sensitive to necroptosis, viral replication was attenuated in each M45 RHIM-swap virus infection, indicating that, similarly to M45*mut*RHIM MCMV, M45 RHIM-swap MCMVs are unable to block necroptosis, thus allowing for the clearance of infection. Finally, when cell death was assessed in cell lines containing necroptotic machinery, M45 RHIM-swap viruses induced ZBP1-dependent necroptosis in both end-point cell-death assays as well as real-time kinetic analyses of cell death. Together, these results indicate that the 16 amino-acid sequences of the two RHIMs participating in the RHIM–RHIM interaction are important in dictating whether a RHIM–RHIM interaction will be pro-survival or pro-death. The results above clearly identify a schism: the ability for the M45 RHIM-swap proteins to bind to RIPK3 does not confer protection from necroptotic cell death in the context of either transient transfection or viral infection. The data indicate that M45 binding to RIPK3 is separable from its ability to protect from necroptotic cell death.

We first sought to determine if there were any behavioral differences between true RHIMs (i.e., RHIMs that bind other RHIMs) when placed in the context of M45. Given that host-encoded RHIM-containing proteins promote cell death, whereas virally encoded RHIM-containing proteins block cell death in a species-specific manner, we hypothesized that the sequences of the two RHIMs participating in the RHIM–RHIM interaction played a major role in dictating cell fate. In support of this hypothesis, ICP6 interaction with human RIPK3 is pro-survival, whereas ICP6 interaction with mouse RIPK3 induces necroptosis independently of upstream RIPK3 activation [20]. Therefore, it was expected that M45 RHIM-swap proteins that contain murine RHIMs from proteins that propagate death would behave like a pro-death RHIM-containing protein, nucleating death-inducing complexes, similarly to ICP6 interactions with murine RHIMs. Additionally, M45 (1-277) RHIM-swap proteins that contained true RHIMs both bound RIPK3 in a transient transfection assay and formed nuclear puncta, similarly to WT M45 (Figure 1B–D). In support of homo-amyloid-RHIMs forming fibrils that appear as puncta via microscopy, RIPK3 has been shown to form distinct cytosolic puncta that are abrogated when M45 is introduced [29]. In an in vitro FACS-based TNFα cell-death assay, despite being able to bind to RIPK3 and unlike 1-277 WT M45, 1-277 vIRA RHIM-swap proteins were unable to protect cells from necroptosis like M45*mut*RHIM M45 (Figure 1E).

This result is consistent with the literature: although overexpression of WT ICP6 induces death in murine cells, ICP6^M45-RHIM^ is not inherently toxic [25]. The converse swap, M45^ICP6-RHIM^, behaves similarly to WT ICP6, inducing death upon overexpression [25]. Interestingly, our results differ slightly: overexpression of our M45^ICP6-RHIM^ did not kill murine cells. We suspect the difference in results lies in a critical difference between the constructs. The previously published RHIM-swap constructs included the full-length ICP6 and M45 proteins [25] whereas our expression constructs contain only the N-terminus (amino acids 1-277) of M45, which lacks the C-terminal R1 domain. Both the N-terminal RHIM-containing domain and the C-terminal R1 domain are necessary for ICP6 to nucleate necroptotic death in the context of overexpression; this explains why our M45^ICP6-RHIM^ construct does not possess inherent toxicity [25]. ICP6 forms oligomers via its C-terminal domain and recruits RIPK3 via its N-terminal RHIM domain to nucleate necroptosis in murine cells, suggesting that ICP6 oligomers are necessary to bring together multiple RIPK3 molecules to seed RIPK3 activation [25]. Interestingly, the M45 C-terminal domain does not cause the nucleation of death-inducing complexes in either human or mouse cells and instead provides protection from TNFα-induced necroptosis in human cells [20,25]. This difference between ICP6 and M45 could be due to differences between the RR domains: M45’s RR domain is catalytically inactive [22]. The M45*mut*RHIM mutation and the analogous ICP6^AAAA-RHIM^ mutation abrogate the ability to interact with other RHIM-containing proteins and prevent these proteins from inhibiting necroptosis in the appropriate species, indicating that inhibition of necroptosis in their respective species of origin requires M45*mut*RHIM and ICP6 to bind other RHIM-containing proteins [19,20].

However, our present data, along with previously published work by others, suggest that the mechanism of RHIM-based protection from viral-induced necroptosis is more complex than the ability to bind and that the specific amino-acid sequences of, and thus structures formed by, each RHIM participating in the interaction dictate cell fate [25,27,28,29,30,31,32]. vIRA as well as human and murine RIPK3 and RIPK1 each form distinctive homo-amyloid and hetero-amyloid structures [27,28,29,30,31,32], which supports the hypothesis that vIRA prevents necroptosis by sequestering RIPK3 in an alternative amyloid structure that does not permit either RIPK3 autophosphorylation or activation of MLKL. In support of the model that the fibril structures themselves, as determined by each unique RHIM, drive signal transduction and outcome, the vIRA and RIPK3 complex requires a hetero-RHIM interface in order to prevent necroptosis: vIRA containing the RIPK3 RHIM is unable to prevent necroptotic death in cells expressing WT RIPK3, while cells expressing a RIPK3-vIRA RHIM chimera survive [29].

M45(1-277) protects cells from necroptosis initiated both by the TNFα-RIPK1 and the MCMV-ZBP1 axes [19], but ICP6 requires both its RHIM and its C-terminal RR domain to suppress cell death via the TNFα-RIPK1 axis and only the RHIM domain to suppress necroptosis via the HSV-1-ZBP1 axis [20]. These data highlight an important difference between the proteins [20,21]. However, the possibility remained that the C-terminal RR domain interaction of M45 oligomers could confer protection from death in the context of a natural infection. To test this hypothesis, the RHIM-swap mutations were cloned into the MCMV BAC to generate recombinant MCMV expressing M45 RHIM-swap proteins (Figure 2A–C). These viruses produced levels of M45 similar to those of WT MCMV (Figure 2D) and replicated to levels similar to those of WT MCMV in a multi-step growth curve in cells resistant to necroptosis (Figure 3A), indicating that the M45 RHIM-swap does not affect viral fitness in the absence of necroptosis. However, multi-step growth curves in cells permissive to necroptosis revealed that similarly to M45*mut*RHIM, these M45 RHIM-swap viruses are unable to replicate (Figure 3B,C), suggesting that necroptosis is restricting viral replication.

Finally, cell death was explicitly compared between each virus. In cell lines expressing necroptotic machinery, M45 RHIM-swap MCMVs induced necroptotic cell death similarly to M45*mut*RHIM MCMV, when assessed by both end-point viability assays (Figure 4A,B) and real-time kinetic cell-death assays (Figure 4D,E). Additionally, this was determined to be ZBP1-dependent necroptotic cell death (Figure 4C), which is important because it highlights a distinct difference between ICP6 and M45. In overexpression assays in murine cells, ICP6 nucleates ZBP1-independent necroptotic cell death in a manner requiring both its RHIM and its C-terminal RR domain, whereas M45^ICP6-RHIM^ does not, likely due to differences in the C-terminal domain [21,25]. Here, by showing that M45 RHIM-swap MCMV-induced necroptosis is ZBP1-dependent, we demonstrated that, in the context of infection, these vIRA RHIM-swaps are not directly killing cells. Rather, full-length M45 RHIM-swap constructs are unable to inhibit virus-induced necroptotic death. Since the kinetics of necroptosis in response to each M45 RHIM-swap virus infection are similar to those of M45*mut*RHIM MCMV, it is additionally unlikely that the action of M45 RHIM-swap proteins binding RIPK3 causes them to seed necroptotic death. After infection of murine cells, HSV-1 induces necroptosis via both ZBP1 activation and ICP6 nucleating activation of RIPK3 [21]. As such, HSV-1 deficient in ICP6 or expressing ICP6^AAAA-RHIM^ induces necroptosis with slower kinetics than WT HSV-1, and ZBP1 knockout cells undergo necroptosis in response to infection with WT but not ICP6 deficient or ICP6^AAAA-RHIM^ HSV-1 [21]. Furthermore, ZBP1 knockout cells do not undergo necroptosis in response to infection with vIRA RHIM-swap MCMVs (Figure 4C), highlighting distinctive differences between ICP6 and M45. Although we have not formally demonstrated whether full-length M45 RHIM-swap proteins bind RIPK3 during MCMV infection, we expect that they do, since the M45 (1-277) RHIM-swap constructs bind RIPK3.

Together our data showing that viral replication of M45 RHIM-swap viruses is restricted in cell lines permissive to necroptosis (Figure 3B,C) indicate that the protection from necroptosis offered by M45 is more complex than the simple sequestration of RIPK3. This supports our initial hypothesis that the 16 amino-acid RHIM sequences participating in RHIM–RHIM interactions are responsible for determining cell fate, likely due to the different amyloid fibril structures formed. The exact differences between RHIMs that cause such drastically different outcomes as well as the minimal RHIM required for RIPK3 binding remain to be determined.

## Figures and Tables

**Table 1:** Identifiers and sequences of oligonucleotides and primers used to generate M45 RHIM-swap constructs.

	Primer Name	Sequence
pEGFP/M45	HS38	TAGGGCACGCTGTGCCCCCCACCCACCCCGA
pEGFP/M45	HS39	AGCATCGCCTCTTGCTGGAGTCCCTCCTACACTGACCGA
RIPK1 RHIM	HS40	GGTGGGGGGCACAGCGTGCCCTACGTCAGGACTATATTCAATAGTTCTGGTATTCAGATTGGAAACCACAATTATATG
RIPK1 RHIM	HS41	AGGAGGGACTCCAGCAAGAGGCGATGCTCATATAATTGTGGTTTCCAATCTGAATACCAGAACTATTGAATATAGT
RIPK3 RHIM	HS42	GGTGGGGGGCACAGCGTGCCCTACGTCAGGGTCTTCAACAACTGTTCGAAGTGCAGATTGGGAACTACAACTCCTTG
RIPK3 RHIM	HS43	AGGAGGGACTCCAGCAAGAGGCGATGCTCAAGGAGTTGTAGTTCCCAATCTGCACTTCAGAACAGTTGTTGAAGAC
TRIF RHIM	HS44	GGTGGGGGGCACAGCGTGCCCTACGTCAGGATTATTCACCATGCCCAGATGGTTCAGCTGGGTGTCAACAATCACATG
TRIF RHIM	HS45	AGGAGGGACTCCAGCAAGAGGCGATGCTCATGTGATTGTTGACACCCAGCTGAACCATCTGGGCATGGTGAATAAT
ZBP1-A RHIM	HS46	GGTGGGGGGCACAGCGTGCCCTACGTCAGGTCCATTGCCAATTCAAACGCCATCCAGATTGGTCACGGGAATGTCATA
ZBP1-A RHIM	HS47	AGGAGGGACTCCAGCAAGAGGCGATGCTTATGACATTCCCGTGACCAATCTGGATGGCGTTTGAATTGGCAATGGA
PARP12 RHIM	HS48	GGTGGGGGGCACAGCGTGCCCTACGTCAGGGCCCAGGCAGCCACCTTGAAGTTCCAGGCTGGAAAACACAACTATGAG
PARP12 RHIM	HS49	AGGAGGGACTCCAGCAAGAGGCGATGCTCTCATAGTTGTGTTTTCCAGCCTGGAACTTCAAGGTGGCTGCCTGGGC
ICP6 RHIM	HS50	GGTGGGGGGCACAGCGTGCCCTACGTCAGGCGCATCAGCGATAGCAACTTTGTCCAATGTGGTTCCAACTGCACCATG
ICP6 RHIM	HS51	AGGAGGGACTCCAGCAAGAGGCGATGCTCATGGTGCAGTTGGAACCACATTGGACAAAGTTGCTATCGCTGATGCG
ZBP1-B RHIM	HS66	GGTGGGGGGCACAGCGTGCCCTACGTCAGGGACAAGTCCTTGCTCCAACAAGTGCAGCTTGGCCACCACAACGAGATG
ZBP1-B RHIM	HS67	AGGAGGGACTCCAGCAAGAGGCGATGCTCATCTCGTTGTGGTGGCCAAGCTGCACTTGTTGGAGCAAGGACTTGTC

**Table 2:** Identifiers and sequences of primers used to sequence recombinant RHIM-swap MCMVs.

	Primer Name	Sequence
Sequencing M45 RHIM locus	HS36	CCCAAAGTGTACTCCGACCC
Sequencing M45 RHIM locus	HS37	GCTTCTTGGCTTGAGGTGC
RHIM amplification	ASP01C	CAGCTCTCCGTGGTT
RHIM amplification	ASP01B	AGCGGCAGGTGAGGA

## Data Availability

The raw data supporting the conclusions of this article will be made available by the authors on request.
